# Microbial Inoculants and Their Impact on Soil Microbial Communities: A Review

**DOI:** 10.1155/2013/863240

**Published:** 2013-07-11

**Authors:** Darine Trabelsi, Ridha Mhamdi

**Affiliations:** Laboratory of Legumes, Centre of Biotechnology of Borj-Cédria, P.O. Box 901, 2050 Hammam-Lif, Tunisia

## Abstract

The knowledge of the survival of inoculated fungal and bacterial strains in field and the effects of their release on the indigenous microbial communities has been of great interest since the practical use of selected natural or genetically modified microorganisms has been developed. Soil inoculation or seed bacterization may lead to changes in the structure of the indigenous microbial communities, which is important with regard to the safety of introduction of microbes into the environment. Many reports indicate that application of microbial inoculants can influence, at least temporarily, the resident microbial communities. However, the major concern remains regarding how the impact on taxonomic groups can be related to effects on functional capabilities of the soil microbial communities. These changes could be the result of direct effects resulting from trophic competitions and antagonistic/synergic interactions with the resident microbial populations, or indirect effects mediated by enhanced root growth and exudation. Combination of inoculants will not necessarily produce an additive or synergic effect, but rather a competitive process. The extent of the inoculation impact on the subsequent crops in relation to the buffering capacity of the plant-soil-biota is still not well documented and should be the focus of future research.

## 1. Introduction

The application of inoculants is seen as being very attractive since it would substantially reduce the use of chemical fertilizers and pesticides, and there are now an increasing number of inoculants being commercialized for various crops [1]. Microorganisms play an important role in agricultural systems, particularly plant growth-promoting microorganisms (PGPMs). Plant growth benefits may be attributed mainly to three mechanisms as follow. (i) PGPMs acting as biofertilizers (such as nitrogen-fixing bacteria and phosphate-solubilizing bacteria) assist plant nutrient uptake by providing fixed nitrogen or other nutrients [2]. (ii) Phytostimulators (microbes expressing phytohormones such as *Azospirillum*) can directly promote the growth of plants, usually by producing plant hormones [3, 4]. (iii) Biological control agents (such as *Trichoderma*, *Pseudomonas,* and *Bacillus*) protect plants against phytopathogenic organisms [5–7]. Several reviews have discussed various aspects of growth promotion by PGPMs [8–10]. However, the potential environmental impacts related to inoculation were always neglected. Since inoculation consists in supplying high densities of viable and efficient microbes for a rapid colonization of the host rhizosphere, it would induce at least a transient perturbation of the equilibrium of soil microbial communities. Changes in microbial composition may be undesirable if important native species are lost, thus affecting subsequent crops. However, a modification in the bacterial community structure caused by inoculation could be buffered by ecosystem resilience, which is driven by the level of diversity and interactions of the plant-soil-biota [11]. The loss of certain bacterial species may however not change the functioning of the system because of the bacterial redundancy, since different bacterial species may carry out the same functions [11, 12].

Soil microbial community is complex and dynamic and varies in composition between different compartments and levels, which represents a real challenge in soil ecology. One of the crucial problems to face in this type of studies is the representativeness of the sampling. Number of replicates, sample size, whether sampling is randomized or at regular intervals, spatial scaling, and microsite variation remain major concerns. Most researchers used rhizospheric soil, but even in this case it is practically very difficult to define it precisely. However, the side-distance effect on bulk soil would be more reliable to address a more generalized response. Time-course studies would be also necessary to monitor inoculation effect in relation to the buffering capacity of the ecosystem. Nevertheless, the techniques used to investigate soil microbial communities at taxonomic and functional levels are laborious and limit the use of exhaustive samplings. In the culture-dependent methods, the analysis is usually confined to restricted samples, and a biased image is drawn. However, the culture-independent methods do not usually permit unambiguous identification of taxonomic groups [13]. Besides the bias induced by DNA extraction and PCR amplification, the culture-independent methods represent also some inherent limitations (Table 1).

The high-throughput sequencing techniques, even being more informative [31, 32], are still not economically affordable for inoculation impact analysis, because of the high number of samples and replicates involved. There is a growing interest in the recent years in genes and transcripts coding for metabolic enzymes. Besides questions addressing redundancy and diversity, more and more attention is given to the abundance of specific DNA and mRNA in the different habitats [33].

## 2. The Rhizosphere: The Unrevealed World

The rhizosphere represents one of the most diverse habitats on the planet and is central to ecosystem functioning. Infinite dynamic interactions between root exudates, microbial activity, genetic exchange, nutrient transformation, and gradient diffusion are most likely the factors shaping this below-ground world (for review, please see [34]). Consequently, there is an increasing need to understand its functioning to effectively manage ecosystem and harness its potential benefits. In particular, manipulation of the rhizosphere is now considered as a key mechanism for solving critical issues facing the planet, including agricultural and forest sustainability, improving water quality, mitigation of climate change, and preservation of biodiversity. To face the range of biotic and abiotic stresses, plants interact with different members of the soil microbial community in many ways and in a complex range of trophic cascades [35]. These relations involve positive and negative feedbacks between soil organisms and plants and their chemical environment. Rhizobacteria communicate within species using diffused chemicals. N-acylhomoserine lactones (N-AHLs) are common signals for bacterial communications in various Gram-negative bacteria [36]. Plant-Microbe interactions are generally regulated by quorum sensing (QS) in a population-dependent manner [37]. Crépin et al. [38] showed that the rhizosphere bacterium *Rhodococcus erythropolis* catabolizes the N-AHLs produced by the pathogenic bacterium *Pectobacterium atrosepticum,* thus attenuating its virulence, which suggests a tritrophic belowground chemical interaction. Pathogens exploit QS using N-AHLs to form microcolonies (and biofilm) in the rhizosphere to inflict pathogenicity in the host. By contrast, beneficial or nonpathogenic microbes biosynthesize N-AHLs degrading lactonases to disrupt QS by quorum-quenching [39]. The presence of biostimulating molecules or QS-mimics may also establish microbes that degrade N-AHLs in the rhizosphere, thus reducing the virulence of pathogens. Another example shows that root volatiles may serve as foraging cues for parasitic entomopathogenic nematodes [40], and, then, poorly available organic phosphorus could be made available through the grazing by nematodes of phytase-producing bacteria [41]. There may be also a synergistic effect of root volatiles and CO_2_ as attractants for nematodes [42]. Kawasaki et al. [43] showed that some legumes respond to certain contaminants in a systemic manner by utilizing root-colonizing microorganisms to protect themselves, and they may actively stimulate such microorganisms in the rhizosphere by manipulation of exudates flux and composition. Surfactant-active compounds in the root exudates increase the solubility of the contaminant and make it more bioavailable to root-colonizing microorganisms [44]. The production of plant growth regulators such as auxin, cytokinin, and gibberellin by PGPMs may also interfere on soil microbial communities through an enhanced root growth and an increased exudation rate [45], without excluding the possibility of a direct effect on the microbial equilibrium leading to the selection of beneficial populations. These few examples illustrate the complexity of the rhizospheric interactions and prove that our knowledge about the plant-soil-biota is just beginning.

## 3. Inoculation Impact Analysis

Different soil microorganisms had been extensively used as inoculants, including rhizobia, *Azospirillum*, mycorrhizal fungi, and biocontrol agents. The most significant studies reporting the effect of these inoculants on soil microbial communities are summarized in Table 2.

### 3.1. Rhizobia Inoculants

Rhizobia are reported to influence crop growth, yield, and nutrient uptake by different mechanisms. They fix nitrogen, help in promoting free-living nitrogen-fixing bacteria, increase supply of other nutrients, such as phosphorus and iron, produce plant hormones, enhance other beneficial bacteria or fungi, control bacterial and fungal diseases, and help in controlling insect pests [8, 10].

Field release of a* Rhizobium etli *strain containing genes encoding trifolitoxin (an antibiotic peptide active against members of a specific group of *α*-proteobacteria that enhances the ability to compete trifolitoxin-sensitive strains) strongly reduced the diversity of trifolitoxin-sensitive members of *α*-proteobacteria in bean rhizosphere as shown by ribosomal intergenic spacer analysis (RISA), with little apparent effect on most microbes [48]. Using a cultivation-dependent approach and a cultivation-independent PCR-single-strand conformation polymorphism (SSCP), Schwieger and Tebbe [14] showed that field release of *Sinorhizobium meliloti* strain L33 affected bacterial diversity in the rhizosphere of alfalfa by reducing the number of *γ*-proteobacteria and increasing the number of *α*-proteobacteria. The shift was interpreted as a replacement of more general bacteria (*Acinetobacter calcoaceticus and Pseudomonas* sp.) by specialists (rhizobia). The indigenous microbial populations in the rhizosphere of alfalfa inoculated with *S. meliloti *strain M403 (a strain with enhanced competitiveness for nodule occupancy constructed by introducing an extra copy of a modified proline dehydrogenase gene) or strain M401 (a control strain carrying the same plasmid without the modified gene) were evaluated by comparing restriction fragment length polymorphism (RFLP) and temperature-gradient gel electrophoresis fingerprints (TGGE) of 16S rDNA genes directly amplified from soil DNA [18]. Seasonal changes were observed in RFLP patterns only when primers specific for *β*- and/or *γ*-proteobacteria were used. RFLP analysis showed that inoculation permitted certain *γ*-proteobacterial populations to be maintained longer and persist in the rhizosphere of alfalfa. Seasonal changes were also detected by TGGE performed with *α*-proteobacterial primers but not with *β*-proteobacterial primers [18]. Therefore, in the TGGE patterns appeared two bands specifically correlated to inoculation with M403, showing sequence similarity, respectively, to *Rahnella aquatilis *and *Kluyvera georgiana*. The seasonal fluctuations induced by environment and alfalfa plants showed much stronger influence on the microbial community than the inoculation-dependent effects.

The quantification of bacterial genes encoding the nitrogenase reductase (*nif*H), ammonia monooxygenase (*amo*A), and nitrite reductase (*nir*K and *nir*S), as well as archaeal *amo*A genes within the nitrogen cycle, was performed in alfalfa rhizosphere inoculated with *S. meliloti* strains S26 and OS6 [26]. At the late flowering phase, a clear correlation was demonstrated between effectiveness of inoculation, as evaluated by nitrogen and carbon contents, and the abundance of *nif*H genes. Moreover, the number of archaeal* amo*A copies increased upon the more effective strain inoculation.

The effects of growth stage, intercropping, and rhizobial inoculation on diversity of soybean endophytic bacteria were demonstrated by the cluster analysis of terminal restriction fragments and the redundancy analysis [49]. The relative abundance in roots was enhanced for *Bradyrhizobium liaoningense* and decreased for *Sinorhizobium americanum*, demonstrating that the endophytic *Sinorhizobium* might be suppressed by competition from the introduced strain. The difference in abundance of endophytic *Sinorhizobium*, *Bradyrhizobium,* and *Rhizobium* in the soybean roots could be correlated to the nodulation autoregulation system of legumes, which is capable of sensing and responding to the presence of inefficient rhizobia by applying sanctions to inactive strains via active control of the permeability of root cortical cells to oxygen, thus limiting the growth of inefficient nodules [20]. A 517 bp TRF assigned to Comamonadaceae and Burkholderiales showed a higher abundance in inoculated soybeans [49]. The abundance of rhizosphere Comamonadaceae bacteria has been previously reported to be associated with mycorrhization [50], and, on the other hand, the latter could be stimulated by the rhizobial inoculation of soybean [51].

In our lab, the effect of on-field inoculation of *Phaseolus vulgaris* with two local rhizobial strains was addressed by exploring community structure and diversity using similarity analysis of T-RFLP profiles. Effects on bacterial community structure and diversity were clearly observed in the bulk soil in the neighborhood of 25 cm of the roots [22]. Both *α*- and *γ*-proteobacteria together with Firmicutes and Actinobacteria were enhanced by inoculation. The mono- and dual inoculation with *Rhizobium gallicum *strain 8a3 and *Ensifer meliloti *strain 4H41 induced the proliferation of bacterial communities that had been frequently reported as PGPMs, like *Rahnella*,* Bacillus*, *Azospirillum*, *Mesorhizobium, Pseudomonas*, *Streptomyces, *and *Sinorhizobium*, among others [23]. The extent of these changes was also seen in the next rotation crop as indicated by the 32% increase observed in potato yield, and also by the 56% decrease in potato wireworm infestation [23]. The number of TRFs is significantly higher in the inoculated treatments than that in the nitrogen-fertilized treatment during flowering and harvesting stages. Similarly, there was a clear trend of increase in intersample heterogeneity of bacterial communities in the inoculated treatment up to the harvesting stage. These data suggest that the perturbation of the community due to inoculation with a rhizobial strain is higher than that due to chemical fertilization and that the evolution of the community in response to inoculants is someway more stochastic, suggesting that the introduction of exogenous bacteria in a community is likely to produce more long-term unpredictable effects than organic nitrogen supply.

### 3.2. *Azospirillum* Inoculants

The agronomic benefits of *Azospirillum* are well documented [52, 53]. Several modes of action are implicated [45], especially the synthesis of phytohormones such as indole-3-acetic acid [3, 54]. Since *Azospirillum* inoculation can have a great effect on root development and exudation [53, 55], it is likely that the use of these phytostimulatory PGPMs will also modify the resident microbial community structure of the rhizosphere. Using the automated ribosomal intergenic spacer analysis (ARISA), inoculation of maize with *Azospirillum lipoferum* CRT1 was seen to increase the variability in the genetic diversity of the rhizobacterial communities between individual field-grown maize plants and between sampling times without modifying the total number of root bacteria [19, 20]. In other studies, the plant growth development was improved with *Azospirillum brasilense* Sp245 by production of auxins, cytokinins, and gibberellins [56]. Root physiology and architecture changed, and root surface area increased through production of more root hairs, leading to an increase in mineral uptake. However, no prominent effects were observed on bacterial communities of maize grown in two different soils and in different growth systems as indicated by DGGE and ARISA [21]. Naiman et al. [28] showed also that inoculation with *Azospirillum* and *Pseudomonas *may exert different effects on the number of specific subgroups of cultivable bacteria in the rhizosphere of wheat. Inoculation changed the profiles of carbon source (CS) utilization of the soil microflora at tillering and grain-filling stages. The CS utilization is related on one hand to the number of microorganisms which are able to use each CS as a sole carbon supply. on the other hand, the importance of growth is a reflection of the functional potential of the community.

Inoculation with two *A. brasilense* strains (40M and 42M) isolated from maize roots changed also the community-level physiological profiles (CLPPs) of the cultivable microbial communities associated with rice [29]. Although the average values of absorbance for arginine showed that the microbial communities associated with inoculated and control plants had significant differences in ability to use arginine, a moderate effect on the physiological and genetic characteristics of microbial communities associated with certain rice cultivars was found under field conditions [24]. The *nif*H T-RFLP-patterns of diazotrophic communities associated with rice roots showed that some of the restriction fragments permitted differentiation of inoculation treatments, such as the 66 bp fragment that could correspond to *Methylogaea oryzae* which is a methanotrophic bacterium associated with rice [24]. These data suggest that the hormonal effect exercised by *Azospirillum* improves the efficiency of the nitrogen absorption leading to superior yields of biomass. The overall rhizosphere diversity, mainly of specific functional groups, is most likely influenced by the change in residual nitrogen rather than the direct effect of inoculation. However, the specific mechanisms causing these changes are still not clear and need to be extensively studied.

### 3.3. Mycorrhizal Fungi Inoculants

Arbuscular mycorrhizal fungi (AMF), grouped into the phylum Glomeromycota [57], have the ability to form mutualistic symbiosis with most land plants and colonize a wider soil volume. They receive carbon from their host, benefiting plant growth through their ability to exploit resources and delivering minerals and water back [58]. AMF affect the soil microorganisms associated with their extraradical mycelium, leading to the formation of a specific zone of soil called the mycorrhizosphere [59, 60]. In the mycorrhizosphere, the AMF might affect negatively [61], positively [62], or may have no effect [63] on microbial biomass and growth of specific microbial taxa [64]. Many studies have shown that some bacterial species respond to the presence of certain AMF [59, 65], suggesting a high degree of specificity between bacteria associated with AMF. Thus, the specific bacteria together with AMF may create more indirect synergism for plant growth [66] including nutrient acquisition [67] and enhancement of root branching [68]. AMF may inhibit pathogen proliferation through the formation of a bacterial community that limits the pathogen invasion [69, 70]. *Glomus intraradices* has been shown to affect positively the bacterial and saprotrophic fungal biomass in a root-free sand environment [62]. Using DGGE of 16S rRNA amplicons from total DNA extracts of pea rhizosphere, Wamberg et al. [46] showed that DGGE profiles were relatively similar between AMF-inoculated and AMF-uninoculated treatments. However, *G. intraradices*-inoculated treatments showed suppression of four to five specific bright bands. These types of changes were also studied in the extreme conditions characteristic of mine tailings [71]. Canonical correspondence analysis of DGGE profiles showed that AMF inoculation significantly influenced the development of both fungal and bacterial rhizosphere community structures after two months. It was reported that the changes observed in the mesquite rhizosphere microbial community structure may be either a direct or an indirect effect of the AMF used. One type of direct interaction between the AMF and bacterial populations in the rhizosphere is the stimulation or suppression of one or more susceptible populations [72]. It was shown that the exudates of the extraradical mycelium of *G. intraradices* could significantly inhibit the conidial germination of the plant pathogen *Fusarium oxysporum* in transformed carrot roots [73]. By contrast, the PGPM *Pseudomonas chlororaphis *was strongly stimulated. One type of indirect effects of *G. intraradices* is the induction of systemic resistance against the parasitic nematodes *Radopholus similis* and *Pratylenchus coffeae* in banana plants [74]. Lioussanne et al. [47] showed that direct root colonization with either *G. mosseae* or *G. intraradices* significantly modified the DGGE bacterial community structure of tomato rhizosphere. The two AMF species had similar bacterial communities after four weeks. The bacterial taxa associated with the rhizosphere of tomato plants inoculated with *G. mosseae* were identified as *Pseudomonas*, *Herbaspirillum,* and *Acidobacterium*, while a *Bacillus simplex* (clone TR03) was found to be affiliated only with *G. intraradices*. One clone (TR2) that was ambiguously identified as *Pseudomonas entomophila*, *Pseudomonas plecoglossicida,* or *Pseudomonas putida* has been associated with both *G. intraradices* and *G. mosseae*. Taken together, all these works showed that AMF can support modification in microbial community structure within mycorrhizosphere. The identification of key microbial populations that are correlated with improved biomass production will help to understand the role of the microbial community in supporting plant growth, suppressing plant pathogen invasion, and other AMF functional abilities.

### 3.4. Biocontrol Inoculants

Most of the commercial rhizobacterial products have been marketed for biological control of plant diseases rather than augmenting plant nutrition or minimizing abiotic stress impacts [1]. A number of microorganisms such as *Trichoderma harzianum* [5, 75], *Pseudomonas fluorescens* [6], and *Bacillus subtilis* [7] have demonstrated antagonism against diseases caused by *Fusarium* spp., *Pythium* spp., *Rhizoctonia* spp., *Sclerotium* spp., and so forth, leading to enhancement in plant growth or yield. It has been established also that application of *B. subtilis* [75], *Pochonia chlamydosporia* [76, 77], and *P. fluorescens* [78–80] can effectively control the diseases caused by nematodes.

Two species of *Pseudomonas* showing antagonistic activity against the tomato pathogen *Ralstonia solanacearum* [27] were assessed for their impacts on the rhizosphere microbial community structure of maize. Shifts in fatty acid methyl ester (FAME) profiles of cultivable bacteria fractions, as well as total rhizosphere microbial communities, were determined in answer to inoculation. The introduced *P. chlororaphis* IDV1 and *P. putida* RA2 showed good survival in the maize rhizosphere. However, both inoculants revealed a small growth-reducing effect towards maize, probably due to changes in the indigenous rhizosphere communities. The community structure transitorily shifted following domination of specific slow-growing bacterial classes. The cultivable fraction of bacteria was represented by high biomass of Gram-positives (i.e., *Bacillus* and *Arthrobacter*) as indicated by the contents of branched FAMEs (especially iso/anteiso 15:0/17:0). During the later growth stage of maize, this group was disturbed by the introduced strains. Actinomycetes, as indicated by fatty acids with a methyl group at carbon 10 of the chain [81], could be supported by young roots of maize, but this effect was inhibited by inoculation [27]. Both *P. chlororaphis* IDV1 and *P. putida* RA2 survived at relatively high density after release, suggesting their direct impact on community structure. In addition, the inoculation shift from Gram-positive-dominated community to more Gram-negative populations might be indicative of a progressive change from oligotrophic to more copiotrophic conditions [82], which could be due to the treatment effect (i.e., nutrients released from dead cells) [27].

To assess the impact of intentionally applied microorganisms on several key ecological aspects of the microbial decomposer community in plant litter, wheat straw on pans of soil was inoculated with either the fungus *Limonomyces roseipellis* or the bacterium *P. fluorescens *strain Pf-5, biocontrol agents for *Pyrenophora tritici-repentis *[83]. *Pseudomonas fluorescens *had insignificant effect, but *L. roseipellis *had measurable effects on some aspects of microbial community structure and function. *Limonomyces roseipellis *altered the frequency distribution of fungal taxa on straw by increasing yeasts and decreasing saprophytic fungi such as *Alternaria *and *Cladosporium*. Compared with nontreated straw, respiration under conditions of adequate moisture (−0.1 MPa) was increased by *L. roseipellis, *but it was unaffected at water potential of −7 MPa. The spectrum of sole carbon sources utilized by the straw microflora was slightly altered in *Limonomyces-treated *straw [83]. This change in metabolic capabilities is attributed to changes in microbial community.

The effects of *Douglas fir* (*Pseudotsuga menziesii*) co-inoculation with the mycorrhiza helper bacterial strain *P. fluorescens* BBc6R8 and/or the fungal strain *Laccaria bicolor* S238N on the indigenous bacterial and ectomycorrhizal communities were assessed using quantitative and qualitative approaches [84]. The inoculated bacterial strain BBc6R8 was not detected after 4 years in any of the treatments where it was speculated that the lack of bacterial effect on seedling growth was due to the nonpersistence of the inoculated bacteria. When *P. fluorescens* BBc6R8 was introduced at sowing, Frey-Klett et al. [85] showed a positive effect of bacterial inoculation on the *Douglas fir*-*L. bicolor* symbiosis despite the bacteria only surviving for up to 19 weeks in the glasshouse and field soil. Heinonsalo et al. [84] suggested that the absence of positive bacterial effect could be due to the too late inoculation of bacteria on already mycorrhized seedlings and that the mycorrhiza helper bacteria promoted the pre-symbiotic survival of the fungus in the soil. Transient effects on soil microbial communities were found following the inoculation with biocontrol agents, such as *P. fluorescens* [86], *Streptomyces melanosporofaciens* [87], and *Corynebacterium glutamicumin* [88]. In a recent work, the effect of *P. fluorescens* 2P24 on soil fungal community in cucumber rhizosphere was detected with T-RFLP and DGGE [25]. The results showed that inoculation had a transient significant effect on soil fungal community, indicating that the period of validity of the biocontrol agent may be limited. All of these results suggest that inoculation with biocontrol agents breaks the original ecological balance of soil microbial community; however, a progressive recovery is commonly observed following fading of the released strain. The effects on soil microbial communities seem more pronounced and more maintained in case of biofertilizers and phytostimulators having direct effects on plants. It is likely that plants amplify and contribute in maintaining the observed effect. Root colonization is certainly a key step in this process.

## 4. Coinoculation versus Monoinoculation

Most inoculants often rely on application of a single strain which might partially account for the recorded inconsistencies in field. A way to overcome this problem is to include different species or strains of beneficial microbes in the same microbial formulation. Coinoculation would combine different mechanisms without the need for genetic engineering [89], increase plant performance, and enhance the efficacy and reliability of healthy effects on crops [90].

Roesti et al. [15] analyzed the effects of PGPR/AMF inoculations on bacterial community structure of wheat rhizosphere. DGGE analysis showed that inoculants induced a significant modification in the bacterial community structure. However, the type of PGPR consortium had more impact on the bacterial community structure (28.3% of the variance) than the presence of AMF (10.6% of the variance). Even though the PGPR strains used produce the antibiotic 2.4-diacetylphloroglucinol, which is known for its antifungal properties, the AMF growth was not affected, and a synergistic effect of PGPR/AMF coinoculation was observed.

The effect of inoculation of *Pinus pinea *with *Bacillus licheniformis *CECT 5106 and *Bacillus pumilus *CECT 5105 was evaluated [91]. Both *Bacillus *strains promoted the growth of *P. pinea *seedlings (probably by gibberellin production), but this biological effect was absent when both strains were coinoculated, indicating a competition effect. The phospholipid fatty acids composition was also altered, despite the low levels of inoculants found at the end of the assay [91].

Also, the combination of *B. subtilis* and *A. brasilense* did not show synergistic or comparable effects on tomato growth comparing with their single applications. Rather, the mutual presence of these microorganisms, other than reducing plant growth, could cause root-architectural alterations [16]. Our results showed also [23] a reduced efficacy of dual inoculation comparing with single inoculations with two rhizobial strains. TRF composition was more diverse in the case of single inoculations. Some of these differentiating TRFs could be affiliated to the genera known as anaerobic nitrogen-fixing consortia and phytohormone producers (i.e., *Clostridium, Bacillus, Stenotrophomonas, *and* Xanthomonas*), which may explain the difference in plant growth. Therefore, combination of inoculants will not necessarily produce an additive or synergic effect, but rather a competitive process, and, hence, growth enhancement could be reduced or eventually disappear. Consequently, the effects on soil microbial communities will be also unpredictable.

## 5. Conclusion

Microbial inoculation may cause tremendous changes in the number and composition of the taxonomic groups. However, the observed impacts depend largely on the techniques used to address the dynamics of soil microbial communities. Some works showed no effect or a transient effect; however, others evidenced a long-term effect. These changes may influence plant and soil and thereby induce unpredictable feedback reactions. Effects on plant growth and protection are not necessarily resulting from a direct effect of the inoculated strain and may be related to induction or repression of resident microbial populations. There may be also synergistic/antagonistic interactions between target and nontarget effects. These changes could well lead to changes in beneficial soil functions such as nitrogen fixation or N-cycling bacteria. The extent of these changes on soil biology is still not well documented and needs to be further assessed. The major concern remains regarding how the impact on taxonomic groups can be related to effects on functional capabilities of the soil microbial communities. The dynamics of these effects in relation to the host crop, the side-distance effect, the mid-term and long-term effects, the crop-rotation effect, and site variation are still not understood and need to be further investigated. Undesirable growing conditions, such as biotic and abiotic stresses, most likely contribute to inconstancy of results and further complicate the problem, but they should be expected as a normal functioning of agriculture. With the evolution of the DNA-sequencing techniques and their accessibility for many working groups, more light will be shed on the complexity of the metabolic potentials of soil microbial communities and their importance to soil ecosystem.

## Figures and Tables

**Table 1:** Advantages and limitations of the culture-independent methods usually used to investigate soil microbial communities.

Method	Advantages	Limitations
SSCP (single-strand conformation polymorphism) [14]	Separates amplified 16S ssDNA by sequence-dependent higher-order structure; great simplicity and speed; automation possible. No GC-clamp is necessary and no gradient gels.	Formation of heteroduplexes; limited phylogenetic information; only short fragments (between 150–400 nucleotides) can be optimally separated. High rate of reannealing during electrophoresis; biases introduced by conformation variations.

DGGE (denaturing-gradient gel electrophoresis) [15–17]	Separates amplified molecules by %GC content on a denaturing gradient; excellent and effective to follow changes of microbial communities in time and space; well suited for monitoring complex communities dominated by a few members; allows phylogenetic identification through excision and sequencing of individual bands.	Limited to dominant communities; samples with high levels of diversity are difficult to resolve; phylogenetic information is limited to bands that are able to be removed and sequenced. A single band does not always mean a single strain; needs careful calibration; limited to DNA fragments typically below 500 bp in size.

TGGE (temperature-gradient gel electrophoresis) [18]	Separates amplified molecules by %GC content on a temperature gradient; operates on the same principle as DGGE; taxonomic information could be obtained from isolated bands.	Provides approximately the same degree of specificity as DGGE and possesses the same advantages and limitations.

RISA (ribosomal RNA intergenic spacer analysis) [19–21]	Amplifies the prokaryotic ribosomal intergenic region creating a community profile based on the species-specific length polymorphisms in this region; great simplicity and speed; automation is possible.	The preferential amplification of shorter sequences is a particular concern; biases imposed by secondary structures in the rDNA genes flanking the amplified region may also pose a problem; small database.

T-RFLP (terminal restriction fragment length polymorphism) [22–25]	Separates amplified and digested molecules according to their length; only terminal restriction fragments (TRFs) are detected and used for qualitative and quantitative analysis; powerful tool for assessing diversity and structure of complex microbial communities; Enables high-throughput and low-cost analysis.	Technical problems arise from inherent pitfalls in the databases used for phylogenetic analysis; overestimation of diversity created by incomplete digestion of environmental DNA and the formation of pseudo (TRFs).

Real-time PCR [26]	Assesses total microbial communities using probes targeting functional genes; the proportions of specific phylotypes can address metabolic potential of the microbial biomass; has superior sensitivity and is more convenient and less expensive for the quantification of selected bacterial populations; quantification of rRNA directly isolated from ribosomes may be used to reveal the metabolically most active members of a bacterial community.	Requires careful calibration; requires extremely accurate controls for inferring cell mass or gene copy; biases introduced by contamination and primer dimers.

MIDI-FAME (Microbial ID, Inc.-fatty acid methyl ester) [27]	Creates a complex profile of fatty acids unique to each community sample; useful in comparing one sample with another and in tracking changes in community structure over time or at different sampling locations.	Quite limited in the taxonomic information; many fatty acids are common to different microorganisms.

CLPP (community-level physiological profiles of carbon sources) [28, 29]	Determine the profile of substrates metabolized by the microbial community; give an estimate of growth and catabolic potential of culturable microorganisms in the original community; relatively inexpensive and commercially are available means of gathering large amounts of information about whole communities of microorganisms.	Long procedure; growth dependent, limited in taxonomic information.

Hybridization arrays (microarrays, beadarrays and Phylochips) [30]	Detect, identify, and potentially quantify thousands of distinct DNA molecules in a single experiment relatively rapidly and cost-effectively; arrays rely on oligonucleotide probes of 16S rRNA from specific groups of organisms to discern phylogeny, community composition, or function; well suited for identification via multiple-gene functional groups.	Limited in exploring entire bacterial diversity. Prior knowledge of the microbial composition is necessary for designing meaningful probes; suffer from cross hybridization between closely related species; genetic variations between strains within species; biases introduced by hairpin structures.

NGS (next-generation sequencing) [31, 32]	The longer reads yield more information about the 16S rRNA and thus give a more accurate identification. Allows accurate comparison between environments; large communities can be studied based on phylogeny and/or function; an average sequence depth of 5000 sequences/sample, up to 200 samples, could be sequenced in parallel.	Intrinsic sequencing errors; overestimation of taxon abundance; primer pairs greatly influence estimates of microbial community richness and evenness; amplicon products are still subjected to the biases inherent to any PCR-based experiment; the reproducibility of amplicon sequencing across a large number of biological replicates is still questionable.

**Table 2:** The most significant studies addressing the impact of inoculation on soil microbial communities.

Inoculant type	Species	Techniques	Major results	
Rhizobia	*S. meliloti* L33	SSCP	The bacterial diversity in the rhizosphere of *Medicago sativa* was qualitatively and quantitatively affected. While the number of members of *γ*-proteobacteria decreased, the number of members of *α*-proteobacteria increased.	[14]
	Cocktail of *Ensifer* strains	DGGE	Field inoculation showed a significant increase of total bacterial diversity due to seasonal changes, but no effect of rhizobial inoculation was observed. DGGE offered little information about bacterial communities.	[17]
	*S. meliloti* M401/M403	TGGE	The persistence of certain *γ*-proteobacterial populations in the rhizosphere of alfalfa could be affected. TGGE proved to be better for identifying specific M403-dependent changes.	[18]
	*R. gallicum* 8a3 *E. meliloti* 4H41	T-RFLP	Field inoculation showed significant effects on bacterial structure and diversity in the bulk soil of common bean. Both *α*- and *γ*-proteobacteria together with Firmicutes and Actinobacteria were enhanced, including beneficial bacterial communities with PGPM potentialities. Dual inoculation was less effective than simple inoculation and induced distinct effects.	[22, 23]
	*S. meliloti* S26/OS6	qPCR	The bacterial genes involved in nitrogen turnover were affected by inoculation. The effectiveness of inoculation was related to the abundance of *nif*H genes in the late flowering phase. A higher number of *amo*A copies were observed during flowering.	[26]

*Azospirillum *	*A. brasilense* Cd/Sp245	DGGE RISA	Field inoculation showed no prominent effects on bacterial communities of maize in two different soils and in different growth systems.	[21]
	*A. lipoferum* CRT1	RISA	Field inoculation of maize increased the intersample variability of the bacterial community between individual plants and sampling times without modifying the total number of root bacteria.	[19, 20]
	*A. brasilense* 40M/42M	CLPPs	Inoculation changed the community-level physiological profiles of the cultivable microbial communities associated with rice roots.	[29]

AMF	*G. intraradices *	DGGE	Inoculation affected the composition of the rhizosphere bacterial community of pea. Four to five specific bands were suppressed. Before flowering, the AMF decreased rhizosphere respiration and number of protozoa, but it did not affect bacterial number. During flowering and pod formation, the AMF stimulated rhizosphere respiration and the negative effect on protozoa decreased.	[46]
	*G. mosseae* *G. intraradices *	DGGE	Inoculation significantly modified the rhizosphere bacterial composition of tomato. The two AMFs had had similar bacterial communities; however, specific species-dependent effects were observed.	[47]

Biocontrol agents	*P. fluorescens* 2P24	T-RFLP DGGE	Inoculation induced a transient effect on fungal community in the rhizosphere of cucumber suggesting that this biocontrol agent has a limited validity. DGGE and T-RFLP showed similar results.	[25]
	*P. chlororaphis* IDV1 *P. putida* RA2	FAME	Inoculation induced shifts in fatty acid methyl ester profiles of cultivable bacteria fractions, as well as total microbial communities in the rhizosphere of maize. The rhizosphere composition shifted from a Gram-positive-dominated community to more Gram-negative populations.	[27]

Coinoculation	*Pseudomonas *spp. AMF	DGGE	Inoculation induced a significant modification in the bacterial community structure. The type of PGPM consortium had more impact on the bacterial community structure than the presence of AMF. A synergistic effect of coinoculation was observed.	[15]
	*B. subtilis* 101 *A. brasilense* Sp245	DGGE	Inoculation with the biocontrol agent did not show significant effects on fungal (18S rRNA) and bacterial (16S rRNA) communities in the rhizosphere of tomato. Combination of the two rhizobacteria had no synergistic or comparable effects on plant biomass, with respect to their single applications.	[16]
	*A. brasilense* *P. fluorescens *	T-RFLP CLPPs	Inoculation did not show significant impact on cultivable communities and *nif*H T-RFLP-patterns of diazotrophic bacteria associated with rice roots. A synergistic effect of coinoculation was found.	[24]
